# Insanity as a Defence in Criminal Cases

**Published:** 1907-02-16

**Authors:** 


### INSANITY AS A DEFENCE IN CRIMINAL CASES.

THAT insanity in some of its forms annuls all criminal responsibility is a well-established doctrine of the common law. The courts for many years have differed only as to the degree of insanity necessary to afford a valid defence. Originally only two kinds of insanity seem to have been recognised by English law—idiocy and lunacy. The law, as laid down by Lord Coke and Lord Hale, and adopted by the courts until the beginning of the last century, declared that to absolve a man from responsibility for his criminal acts his insanity must be such as "totally to deprive him of all memory and understanding." This doctrine has been called the "wild beast" theory, as it inflicted upon an insane person convicted of a capital crime the same punishment it accorded to wild beasts, unless it could be shown that he was possessed of more memory and understanding than they have been allotted by nature. This doctrine received a blow from Mr. Erskine in his defence, on the ground of insanity, of one Hadfield, who was indicted in 1800 for shooting at King George III. in Drury Lane Theatre. Erskine's eloquence and logic gained the day and doubtless obtained the first advance in favour of the insane.

Twelve years later, in the case of Bellingham, tried for the murder of Mr. Spencer Perceval, it was held for the first time that in order to convict the prisoner he must be competent to know the difference between right and wrong in the abstract. This, and Hadfield's case, continued to be the law of England for more than thirty years, although the decisions of the courts were not always in exact accordance with the principles there laid down.

In 1843, McNaughten, a Scotchman, shot a Mr. Drummond, whom he believed to be one of a band of persons following him everywhere, blasting his character, and making his life wretched. He had transacted business a short time before the deed, and had shown no obvious symptoms of insanity in his discourse or conduct. He was, however, acquitted on the ground of insanity. This verdict having caused great public alarm and indignation throughout the kingdom, the House of Lords propounded certain questions to the judges, and their answers constitute the present law of England on the subject (*McNaughten's case*, 10 Cl. and Fin. 200). The substance may be thus stated : "To establish a defence on the ground of insanity it must be clearly proved that at the time of committing the act the party accused was labouring under such a defect of reason, from disease of the mind, as not to know the nature or quality of the act he was doing; or, if he did know it, that he was not aware he was doing what was wrong." Here it will be observed, first, that the question of right and wrong in the abstract was renounced; and, secondly, that now the knowledge of right and wrong, as regards the act in question, was held to be the true test. As to partial insanity, or delusion, as it was termed, the judges said : "That if a person was acting under an insane delusion, and was in other respects sane, he must be considered in the same situation as to responsibility as if the facts, with respect to which the delusion exists, were real. That is to say, that the acts of the criminal should be judged as if he had really been in the circumstances he imagined himself to be in. For example, if under the influence of delusion he supposes another man to be in the act of attempting to take his life, and he kills him, as he supposes, in self-defence, he would be exempt from punishment. If his delusion was that the deceased had inflicted an injury upon him in character and fortune and he killed him in revenge for such supposed injury, he would be liable to punishment."

The answers of the judges have also been generally adopted by the American courts, where, however, there have been many and frequent variations in the application of these answers. Yet it may be said in general, and doubtless it is the common opinion, that on both sides of the Atlantic the test question, in cases where insanity is brought forward as a defence in criminal cases, would be : Did the prisoner, at the time of the commission of the act, have sufficient strength of mind to know the difference between right and wrong in regard to that particular act?

Within the past ten years there has been an important qualification annexed to this ruling in American courts whose opinions are deserving of the highest respect : they have held that not only must the prisoner know the difference between right and wrong as regards the act in question, but also must have sufficient mental power to control the sudden impulses of his own disordered mind. This addition to the rule, as laid down by the English judges, has been of very slow growth, but it is now the well settled law in many of the States.

In Pennsylvania the first decision advancing this doctrine was a case tried in Lycoming county before Chief Justice Lewis. In charging the jury he said, as to "irresistible impulse" : "It is not generally admitted in legal tribunals as a species of insanity which relieves from responsibility for crime, and it ought never to be admitted as a defence until it is shown to exist in such violence as to subjugate the intellect, control the will, and render it impossible for the party to do otherwise than yield. Where its existence is fully established this species of insanity relieves from accountability to human laws." He afterwards repeated the same views in his work on criminal law (Lewis' U.S. Crim. Law, 484).

In *Commonwealth* v. *Mosler* (4 Barr. 264), Chief Justice Gibson said : "His insanity must be so great as entirely to destroy his perception of right and wrong; and it is not until that perception is thus destroyed that he ceases to be responsible. It must amount to delusion or hallucination, controlling his will and making his commission of the act, in his apprehension, a duty of overruling necessity. The law is that whether the insanity be general or partial, the degree of it must be so great as to have controlled the will of its subject and to have taken from him the freedom of moral action." And in *State* v. *Pike* (50 N. H. 441), Judge Doe, another American judge, says : "If the alleged act of the defendant was the act of his mental disease, it was not, in law, his act, and he is no more responsible for it than he would be if it had been the act of his involuntary intoxication, or of another person using the defendant's hand against his utmost resistance. When disease is the propelling, uncontrollable power, the man is as innocent as the weapon—the mental and moral elements are as guiltless as the material. If his mental, moral, and bodily strength is subjugated and pressed to an involuntary service, it is immaterial whether it is done by the disease, or by another, or a brute or any physical force or act of nature set in operation without any fault on his part."

The question now arises : Upon whom rests the burden of proof to show this insanity? Does it devolve upon the prosecution or upon the prisoner? The many varied decisions in the courts of England and the United States render it difficult to present any general rule on the subject. Probably the better established doctrine now is that whenever in the course of the trial evidence is produced showing that the prisoner was of unsound mind, the burden of proof immediately rests on the prosecution to establish the contrary. The onus is first on the prisoner to show that the insanity exists, which being done, it immediately shifts upon the prosecution, and it is for them to show that it does not exist, or, if it does, that it is not such as would prevent him from knowing and doing the right. For the prosecution must not only prove affirmatively that the prisoner committed the deed, but must also show that he was such a free agent as is necessary in the law to make his act a crime. There are several American decisions which decide that the jury are to be governed by the preponderance of the evidence, and that it is not necessary that insanity should be proven beyond a reasonable doubt.

The English rule, on the contrary, holds that the prisoner must prove his insanity beyond a reasonable doubt. The English rule is also the law in New Jersey, Pennsylvania, and North Carolina. This question as to the existence of the insanity, and, if it does exist, if to a sufficient degree to relieve the prisoner from punishment, is purely a matter of fact for the jury, and not of law for the court.

This gradual change in the law of insanity has kept even pace with the treatment offered in the asylums to those afflicted with this disease. The monastic doctrine that the insane were possessed with evil spirits, and were to be treated as criminals, held sway for many years after its founders had lost their control over the minds of men. As the true nature of insanity became known and it was found to be a disease needing care and attention, the treatment of those thus diseased and the laws as to their criminal responsibility became more humane, until to-day we find the insane treated with a degree of kindness and consideration that was unknown at the time such celebrated lawyers as Lord Coke and Lord Hale said their criminal responsibility was to be measured with that of the wild beasts, and when Hogarth gave us a representation of the Bedlam of his day.

